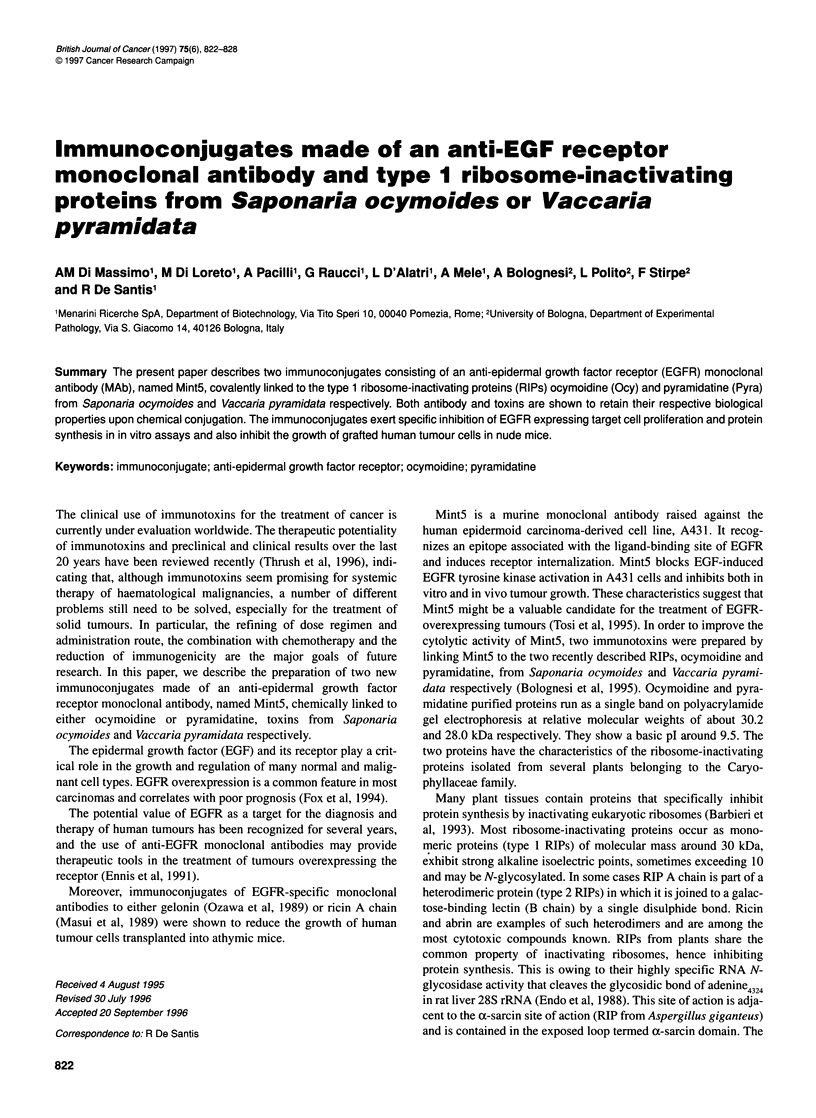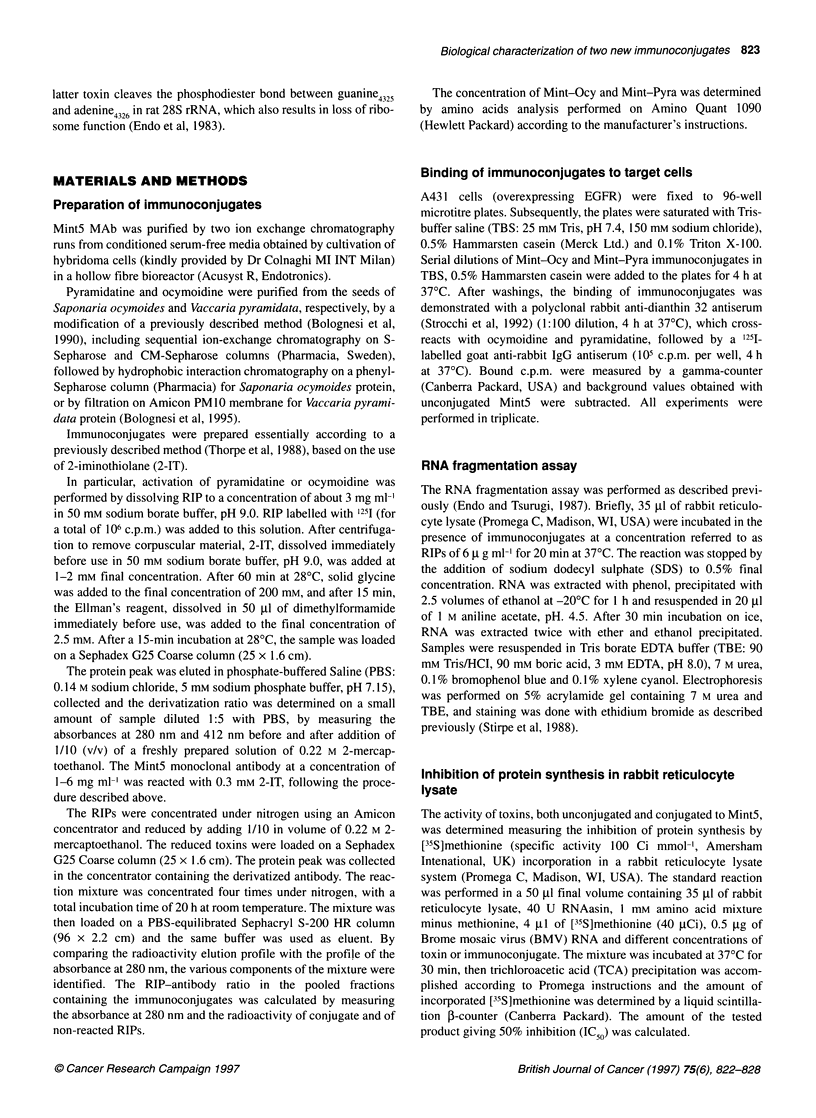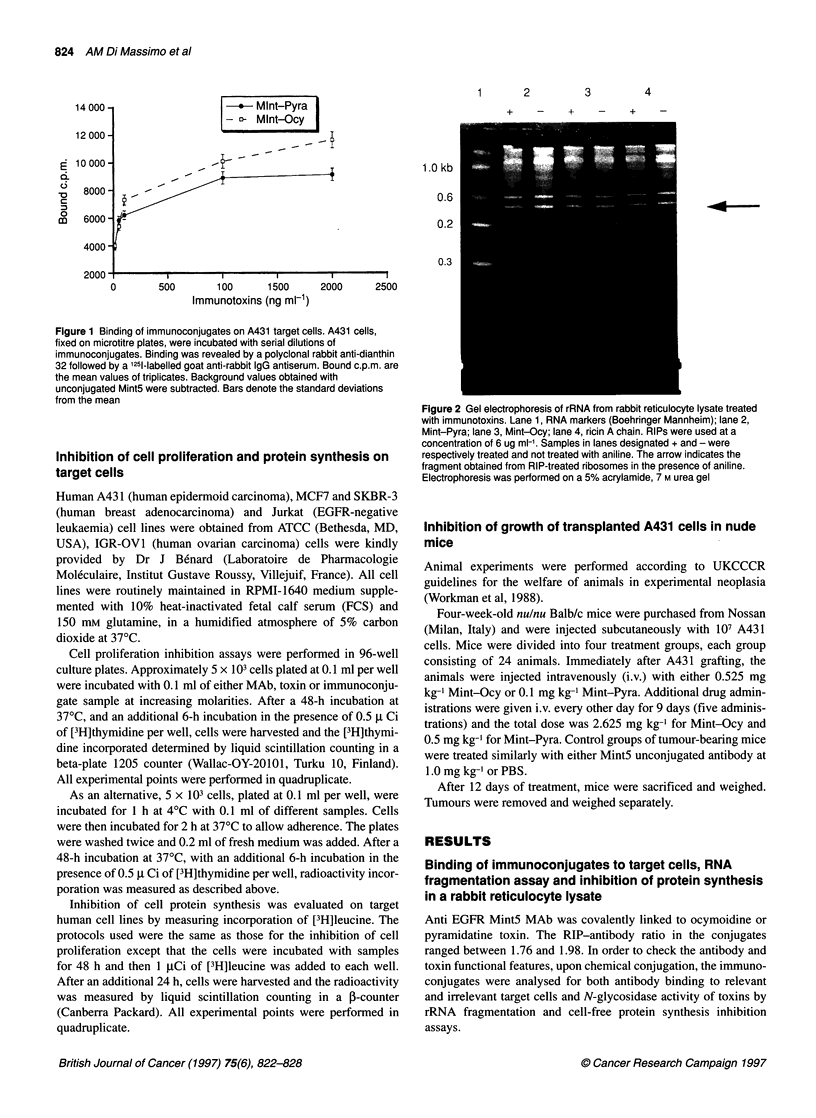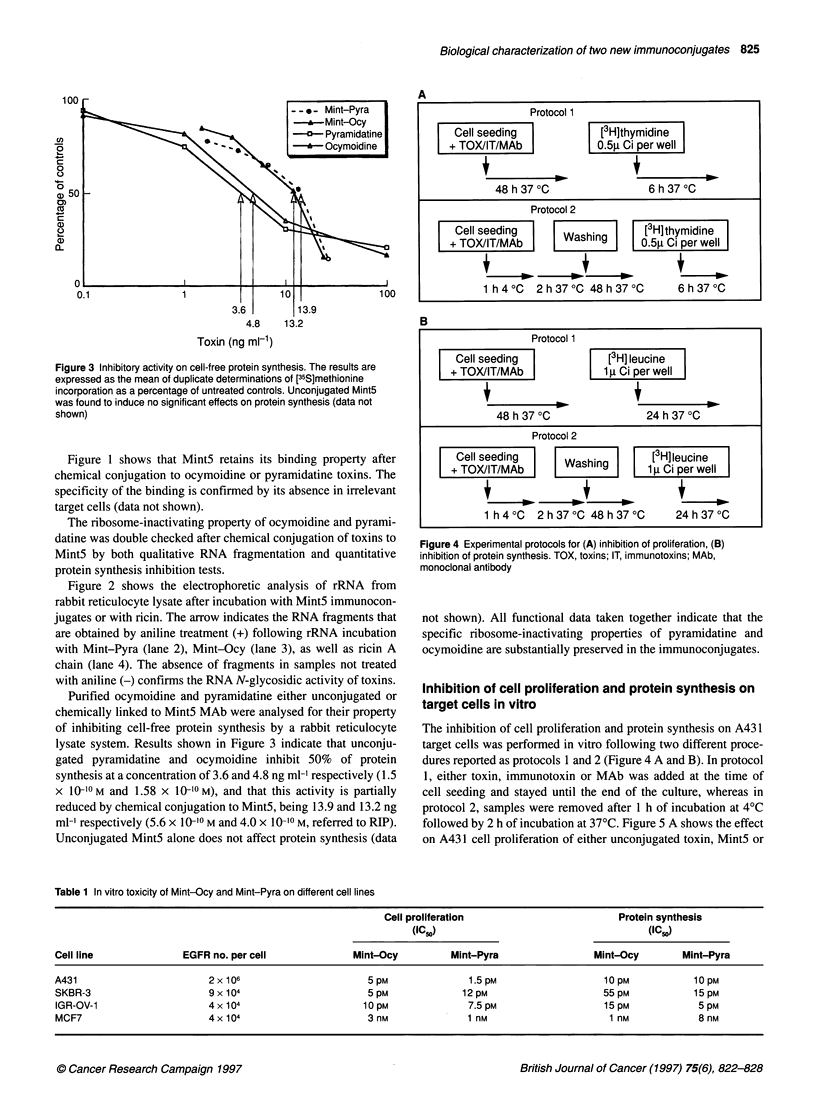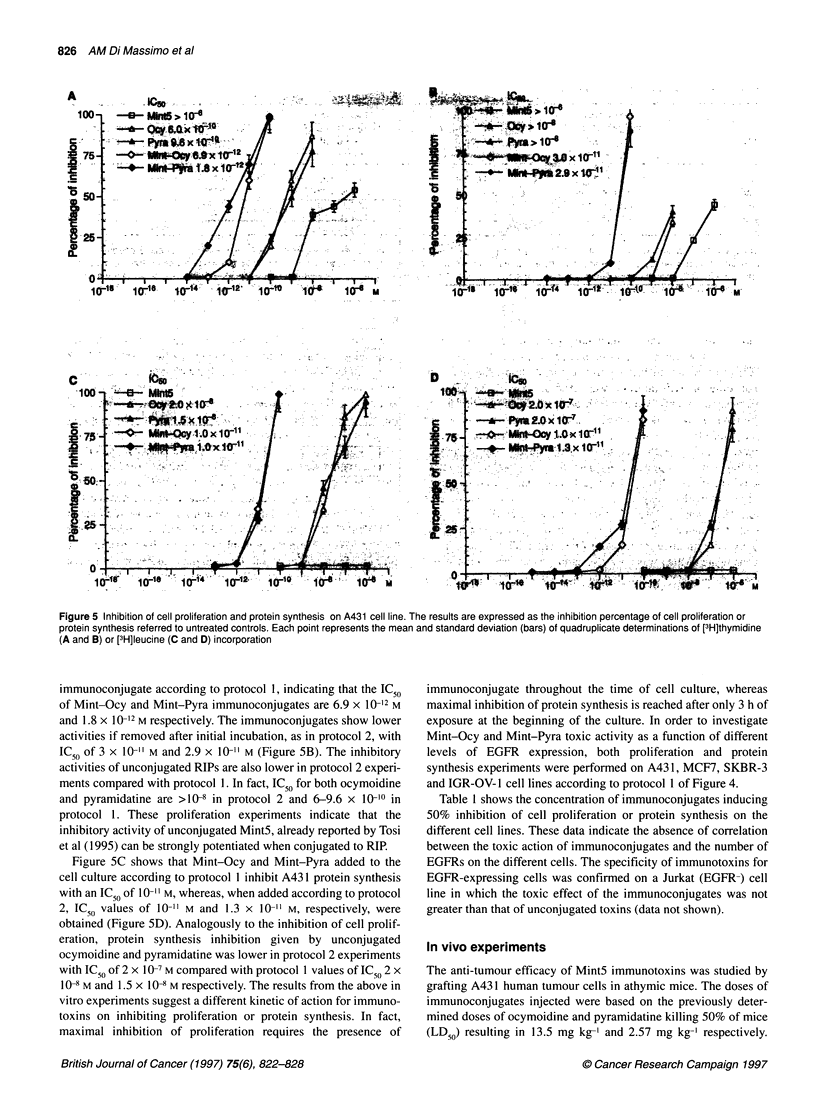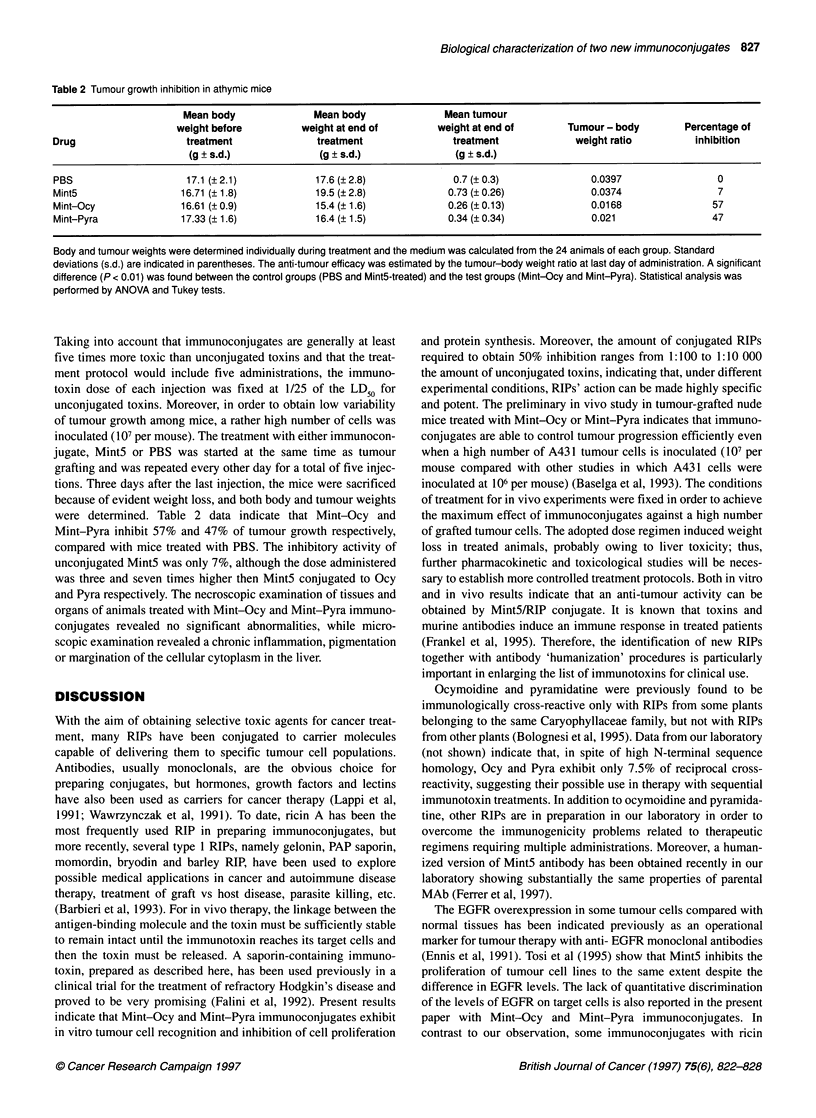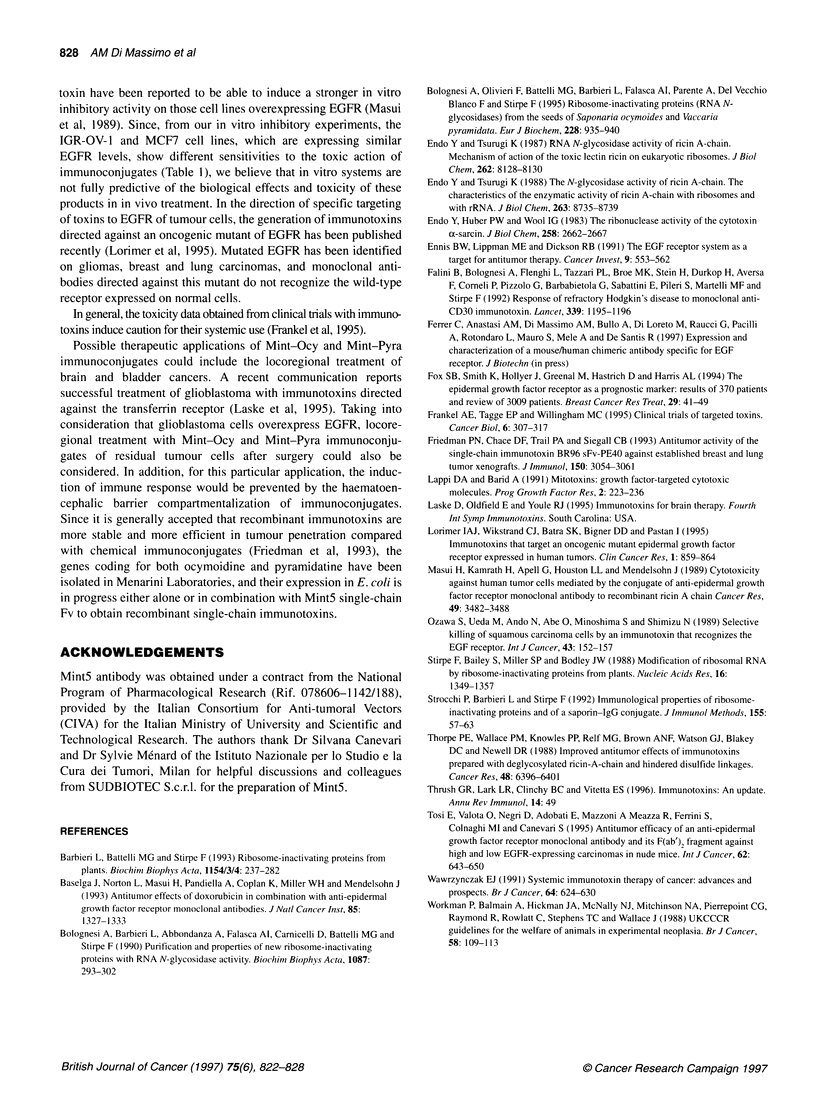# Immunoconjugates made of an anti-EGF receptor monoclonal antibody and type 1 ribosome-inactivating proteins from Saponaria ocymoides or Vaccaria pyramidata.

**DOI:** 10.1038/bjc.1997.147

**Published:** 1997

**Authors:** A. M. Di Massimo, M. Di Loreto, A. Pacilli, G. Raucci, L. D'Alatri, A. Mele, A. Bolognesi, L. Polito, F. Stirpe, R. De Santis

**Affiliations:** Menarini Ricerche SpA, Department of Biotechnology, Pomezia, Rome, Italy.

## Abstract

**Images:**


British Journal of Cancer (1997) 75(6), 822–828
© 1997 Cancer Research Campaign

### Immunoconjugates made of an anti-EGF receptor monoclonal antibody and type 1 ribosome-inactivating proteins from *Saponaria ocymoides* or *Vaccaria pyramidata*

**AM Di Massimo[1], M Di Loreto[1], A Pacilli[1], G Raucci[1], L D'Alatri[1], A Mele[1], A Bolognesi[2], L Polito[2], F Stirpe[2] and R De Santis[1]**

[1]Menarini Ricerche SpA, Department of Biotechnology, Via Tito Speri 10, 00040 Pomezia, Rome; [2]University of Bologna, Department of Experimental Pathology, Via S. Giacomo 14, 40126 Bologna, Italy

**Summary** The present paper describes two immunoconjugates consisting of an anti-epidermal growth factor receptor (EGFR) monoclonal antibody (MAb), named Mint5, covalently linked to the type 1 ribosome-inactivating proteins (RIPs) ocymoidine (Ocy) and pyramidatine (Pyra) from *Saponaria ocymoides* and *Vaccaria pyramidata* respectively. Both antibody and toxins are shown to retain their respective biological properties upon chemical conjugation. The immunoconjugates exert specific inhibition of EGFR expressing target cell proliferation and protein synthesis in in vitro assays and also inhibit the growth of grafted human tumour cells in nude mice.

**Keywords:** immunoconjugate; anti-epidermal growth factor receptor; ocymoidine; pyramidatine

The clinical use of immunotoxins for the treatment of cancer is currently under evaluation worldwide. The therapeutic potentiality of immunotoxins and preclinical and clinical results over the last 20 years have been reviewed recently (Thrush et al, 1996), indicating that, although immunotoxins seem promising for systemic therapy of haematological malignancies, a number of different problems still need to be solved, especially for the treatment of solid tumours. In particular, the refining of dose regimen and administration route, the combination with chemotherapy and the reduction of immunogenicity are the major goals of future research. In this paper, we describe the preparation of two new immunoconjugates made of an anti-epidermal growth factor receptor monoclonal antibody, named Mint5, chemically linked to either ocymoidine or pyramidatine, toxins from *Saponaria ocymoides* and *Vaccaria pyramidata* respectively.

The epidermal growth factor (EGF) and its receptor play a critical role in the growth and regulation of many normal and malignant cell types. EGFR overexpression is a common feature in most carcinomas and correlates with poor prognosis (Fox et al, 1994).

The potential value of EGFR as a target for the diagnosis and therapy of human tumours has been recognized for several years, and the use of anti-EGFR monoclonal antibodies may provide therapeutic tools in the treatment of tumours overexpressing the receptor (Ennis et al, 1991).

Moreover, immunoconjugates of EGFR-specific monoclonal antibodies to either gelonin (Ozawa et al, 1989) or ricin A chain (Masui et al, 1989) were shown to reduce the growth of human tumour cells transplanted into athymic mice.

*Received 4 August 1995*
*Revised 30 July 1996*
*Accepted 20 September 1996*

*Correspondence to:* R De Santis

Mint5 is a murine monoclonal antibody raised against the human epidermoid carcinoma-derived cell line, A431. It recognizes an epitope associated with the ligand-binding site of EGFR and induces receptor internalization. Mint5 blocks EGF-induced EGFR tyrosine kinase activation in A431 cells and inhibits both in vitro and in vivo tumour growth. These characteristics suggest that Mint5 might be a valuable candidate for the treatment of EGFR-overexpressing tumours (Tosi et al, 1995). In order to improve the cytolytic activity of Mint5, two immunotoxins were prepared by linking Mint5 to the two recently described RIPs, ocymoidine and pyramidatine, from *Saponaria ocymoides* and *Vaccaria pyramidata* respectively (Bolognesi et al, 1995). Ocymoidine and pyramidatine purified proteins run as a single band on polyacrylamide gel electrophoresis at relative molecular weights of about 30.2 and 28.0 kDa respectively. They show a basic pI around 9.5. The two proteins have the characteristics of the ribosome-inactivating proteins isolated from several plants belonging to the Caryophyllaceae family.

Many plant tissues contain proteins that specifically inhibit protein synthesis by inactivating eukaryotic ribosomes (Barbieri et al, 1993). Most ribosome-inactivating proteins occur as monomeric proteins (type 1 RIPs) of molecular mass around 30 kDa, exhibit strong alkaline isoelectric points, sometimes exceeding 10 and may be *N*-glycosylated. In some cases RIP A chain is part of a heterodimeric protein (type 2 RIPs) in which it is joined to a galactose-binding lectin (B chain) by a single disulphide bond. Ricin and abrin are examples of such heterodimers and are among the most cytotoxic compounds known. RIPs from plants share the common property of inactivating ribosomes, hence inhibiting protein synthesis. This is owing to their highly specific RNA *N*-glycosidase activity that cleaves the glycosidic bond of adenine$_{4324}$ in rat liver 28S rRNA (Endo et al, 1988). This site of action is adjacent to the α-sarcin site of action (RIP from *Aspergillus giganteus*) and is contained in the exposed loop termed α-sarcin domain. The latter toxin cleaves the phosphodiester bond between guanine$_{4325}$ and adenine$_{4326}$ in rat 28S rRNA, which also results in loss of ribosome function (Endo et al, 1983).

### MATERIALS AND METHODS

### Preparation of immunoconjugates

Mint5 MAb was purified by two ion exchange chromatography runs from conditioned serum-free media obtained by cultivation of hybridoma cells (kindly provided by Dr Colnaghi MI INT Milan) in a hollow fibre bioreactor (Acusyst R, Endotronics).

Pyramidatine and ocymoidine were purified from the seeds of *Saponaria ocymoides* and *Vaccaria pyramidata*, respectively, by a modification of a previously described method (Bolognesi et al, 1990), including sequential ion-exchange chromatography on S-Sepharose and CM-Sepharose columns (Pharmacia, Sweden), followed by hydrophobic interaction chromatography on a phenyl-Sepharose column (Pharmacia) for *Saponaria ocymoides* protein, or by filtration on Amicon PM10 membrane for *Vaccaria pyramidata* protein (Bolognesi et al, 1995).

Immunoconjugates were prepared essentially according to a previously described method (Thorpe et al, 1988), based on the use of 2-iminothiolane (2-IT).

In particular, activation of pyramidatine or ocymoidine was performed by dissolving RIP to a concentration of about 3 mg ml$^{-1}$ in 50 mM sodium borate buffer, pH 9.0. RIP labelled with $^{125}$I (for a total of $10^6$ c.p.m.) was added to this solution. After centrifugation to remove corpuscular material, 2-IT, dissolved immediately before use in 50 mM sodium borate buffer, pH 9.0, was added at 1–2 mM final concentration. After 60 min at 28°C, solid glycine was added to the final concentration of 200 mM, and after 15 min, the Ellman's reagent, dissolved in 50 µl of dimethylformamide immediately before use, was added to the final concentration of 2.5 mM. After a 15-min incubation at 28°C, the sample was loaded on a Sephadex G25 Coarse column (25 × 1.6 cm).

The protein peak was eluted in phosphate-buffered Saline (PBS: 0.14 M sodium chloride, 5 mM sodium phosphate buffer, pH 7.15), collected and the derivatization ratio was determined on a small amount of sample diluted 1:5 with PBS, by measuring the absorbances at 280 nm and 412 nm before and after addition of 1/10 (v/v) of a freshly prepared solution of 0.22 M 2-mercaptoethanol. The Mint5 monoclonal antibody at a concentration of 1–6 mg ml$^{-1}$ was reacted with 0.3 mM 2-IT, following the procedure described above.

The RIPs were concentrated under nitrogen using an Amicon concentrator and reduced by adding 1/10 in volume of 0.22 M 2-mercaptoethanol. The reduced toxins were loaded on a Sephadex G25 Coarse column (25 × 1.6 cm). The protein peak was collected in the concentrator containing the derivatized antibody. The reaction mixture was concentrated four times under nitrogen, with a total incubation time of 20 h at room temperature. The mixture was then loaded on a PBS-equilibrated Sephacryl S-200 HR column (96 × 2.2 cm) and the same buffer was used as eluent. By comparing the radioactivity elution profile with the profile of the absorbance at 280 nm, the various components of the mixture were identified. The RIP–antibody ratio in the pooled fractions containing the immunoconjugates was calculated by measuring the absorbance at 280 nm and the radioactivity of conjugate and of non-reacted RIPs.

The concentration of Mint–Ocy and Mint–Pyra was determined by amino acids analysis performed on Amino Quant 1090 (Hewlett Packard) according to the manufacturer's instructions.

### Binding of immunoconjugates to target cells

A431 cells (overexpressing EGFR) were fixed to 96-well microtitre plates. Subsequently, the plates were saturated with Tris-buffer saline (TBS: 25 mM Tris, pH 7.4, 150 mM sodium chloride), 0.5% Hammarsten casein (Merck Ltd.) and 0.1% Triton X-100. Serial dilutions of Mint–Ocy and Mint–Pyra immunoconjugates in TBS, 0.5% Hammarsten casein were added to the plates for 4 h at 37°C. After washings, the binding of immunoconjugates was demonstrated with a polyclonal rabbit anti-dianthin 32 antiserum (Strocchi et al, 1992) (1:100 dilution, 4 h at 37°C), which cross-reacts with ocymoidine and pyramidatine, followed by a $^{125}$I-labelled goat anti-rabbit IgG antiserum ($10^5$ c.p.m. per well, 4 h at 37°C). Bound c.p.m. were measured by a gamma-counter (Canberra Packard, USA) and background values obtained with unconjugated Mint5 were subtracted. All experiments were performed in triplicate.

### RNA fragmentation assay

The RNA fragmentation assay was performed as described previously (Endo and Tsurugi, 1987). Briefly, 35 µl of rabbit reticulocyte lysate (Promega C, Madison, WI, USA) were incubated in the presence of immunoconjugates at a concentration referred to as RIPs of 6 µ g ml$^{-1}$ for 20 min at 37°C. The reaction was stopped by the addition of sodium dodecyl sulphate (SDS) to 0.5% final concentration. RNA was extracted with phenol, precipitated with 2.5 volumes of ethanol at –20°C for 1 h and resuspended in 20 µl of 1 M aniline acetate, pH. 4.5. After 30 min incubation on ice, RNA was extracted twice with ether and ethanol precipitated. Samples were resuspended in Tris borate EDTA buffer (TBE: 90 mM Tris/HCI, 90 mM boric acid, 3 mM EDTA, pH 8.0), 7 M urea, 0.1% bromophenol blue and 0.1% xylene cyanol. Electrophoresis was performed on 5% acrylamide gel containing 7 M urea and TBE, and staining was done with ethidium bromide as described previously (Stirpe et al, 1988).

### Inhibition of protein synthesis in rabbit reticulocyte lysate

The activity of toxins, both unconjugated and conjugated to Mint5, was determined measuring the inhibition of protein synthesis by [$^{35}$S]methionine (specific activity 100 Ci mmol$^{-1}$, Amersham Intenational, UK) incorporation in a rabbit reticulocyte lysate system (Promega C, Madison, WI, USA). The standard reaction was performed in a 50 µl final volume containing 35 µl of rabbit reticulocyte lysate, 40 U RNAasin, 1 mM amino acid mixture minus methionine, 4 µl of [$^{35}$S]methionine (40 µCi), 0.5 µg of Brome mosaic virus (BMV) RNA and different concentrations of toxin or immunoconjugate. The mixture was incubated at 37°C for 30 min, then trichloroacetic acid (TCA) precipitation was accomplished according to Promega instructions and the amount of incorporated [$^{35}$S]methionine was determined by a liquid scintillation β-counter (Canberra Packard). The amount of the tested product giving 50% inhibition ($IC_{50}$) was calculated.

© Cancer Research Campaign 1997

**Figure 1** Binding of immunoconjugates on A431 target cells. A431 cells, fixed on microtitre plates, were incubated with serial dilutions of immunoconjugates. Binding was revealed by a polyclonal rabbit anti-dianthin 32 followed by a $^{125}$I-labelled goat anti-rabbit IgG antiserum. Bound c.p.m. are the mean values of triplicates. Background values obtained with unconjugated Mint5 were subtracted. Bars denote the standard deviations from the mean

**Figure 2** Gel electrophoresis of rRNA from rabbit reticulocyte lysate treated with immunotoxins. Lane 1, RNA markers (Boehringer Mannheim); lane 2, Mint–Pyra; lane 3, Mint–Ocy; lane 4, ricin A chain. RIPs were used at a concentration of 6 ug ml$^{-1}$. Samples in lanes designated + and – were respectively treated and not treated with aniline. The arrow indicates the fragment obtained from RIP-treated ribosomes in the presence of aniline. Electrophoresis was performed on a 5% acrylamide, 7 M urea gel

### Inhibition of cell proliferation and protein synthesis on target cells

Human A431 (human epidermoid carcinoma), MCF7 and SKBR-3 (human breast adenocarcinoma) and Jurkat (EGFR-negative leukaemia) cell lines were obtained from ATCC (Bethesda, MD, USA), IGR-OV1 (human ovarian carcinoma) cells were kindly provided by Dr J Bénard (Laboratoire de Pharmacologie Moléculaire, Institut Gustave Roussy, Villejuif, France). All cell lines were routinely maintained in RPMI-1640 medium supplemented with 10% heat-inactivated fetal calf serum (FCS) and 150 mM glutamine, in a humidified atmosphere of 5% carbon dioxide at 37°C.

Cell proliferation inhibition assays were performed in 96-well culture plates. Approximately $5 \times 10^3$ cells plated at 0.1 ml per well were incubated with 0.1 ml of either MAb, toxin or immunoconjugate sample at increasing molarities. After a 48-h incubation at 37°C, and an additional 6-h incubation in the presence of 0.5 μ Ci of [$^3$H]thymidine per well, cells were harvested and the [$^3$H]thymidine incorporated determined by liquid scintillation counting in a beta-plate 1205 counter (Wallac-OY-20101, Turku 10, Finland). All experimental points were performed in quadruplicate.

As an alternative, $5 \times 10^3$ cells, plated at 0.1 ml per well, were incubated for 1 h at 4°C with 0.1 ml of different samples. Cells were then incubated for 2 h at 37°C to allow adherence. The plates were washed twice and 0.2 ml of fresh medium was added. After a 48-h incubation at 37°C, with an additional 6-h incubation in the presence of 0.5 μ Ci of [$^3$H]thymidine per well, radioactivity incorporation was measured as described above.

Inhibition of cell protein synthesis was evaluated on target human cell lines by measuring incorporation of [$^3$H]leucine. The protocols used were the same as those for the inhibition of cell proliferation except that the cells were incubated with samples for 48 h and then 1 μCi of [$^3$H]leucine was added to each well. After an additional 24 h, cells were harvested and the radioactivity was measured by liquid scintillation counting in a β-counter (Canberra Packard). All experimental points were performed in quadruplicate.

### Inhibition of growth of transplanted A431 cells in nude mice

Animal experiments were performed according to UKCCCR guidelines for the welfare of animals in experimental neoplasia (Workman et al, 1988).

Four-week-old *nu/nu* Balb/c mice were purchased from Nossan (Milan, Italy) and were injected subcutaneously with $10^7$ A431 cells. Mice were divided into four treatment groups, each group consisting of 24 animals. Immediately after A431 grafting, the animals were injected intravenously (i.v.) with either 0.525 mg kg$^{-1}$ Mint–Ocy or 0.1 mg kg$^{-1}$ Mint–Pyra. Additional drug administrations were given i.v. every other day for 9 days (five administrations) and the total dose was 2.625 mg kg$^{-1}$ for Mint–Ocy and 0.5 mg kg$^{-1}$ for Mint–Pyra. Control groups of tumour-bearing mice were treated similarly with either Mint5 unconjugated antibody at 1.0 mg kg$^{-1}$ or PBS.

After 12 days of treatment, mice were sacrificed and weighed. Tumours were removed and weighed separately.

### RESULTS

### Binding of immunoconjugates to target cells, RNA fragmentation assay and inhibition of protein synthesis in a rabbit reticulocyte lysate

Anti EGFR Mint5 MAb was covalently linked to ocymoidine or pyramidatine toxin. The RIP–antibody ratio in the conjugates ranged between 1.76 and 1.98. In order to check the antibody and toxin functional features, upon chemical conjugation, the immunoconjugates were analysed for both antibody binding to relevant and irrelevant target cells and *N*-glycosidase activity of toxins by rRNA fragmentation and cell-free protein synthesis inhibition assays.

 © Cancer Research Campaign 1997

**Figure 3** Inhibitory activity on cell-free protein synthesis. The results are expressed as the mean of duplicate determinations of [$^{35}$S]methionine incorporation as a percentage of untreated controls. Unconjugated Mint5 was found to induce no significant effects on protein synthesis (data not shown)

Figure 1 shows that Mint5 retains its binding property after chemical conjugation to ocymoidine or pyramidatine toxins. The specificity of the binding is confirmed by its absence in irrelevant target cells (data not shown).

The ribosome-inactivating property of ocymoidine and pyramidatine was double checked after chemical conjugation of toxins to Mint5 by both qualitative RNA fragmentation and quantitative protein synthesis inhibition tests.

Figure 2 shows the electrophoretic analysis of rRNA from rabbit reticulocyte lysate after incubation with Mint5 immunoconjugates or with ricin. The arrow indicates the RNA fragments that are obtained by aniline treatment (+) following rRNA incubation with Mint–Pyra (lane 2), Mint–Ocy (lane 3), as well as ricin A chain (lane 4). The absence of fragments in samples not treated with aniline (–) confirms the RNA *N*-glycosidic activity of toxins.

Purified ocymoidine and pyramidatine either unconjugated or chemically linked to Mint5 MAb were analysed for their property of inhibiting cell-free protein synthesis by a rabbit reticulocyte lysate system. Results shown in Figure 3 indicate that unconjugated pyramidatine and ocymoidine inhibit 50% of protein synthesis at a concentration of 3.6 and 4.8 ng ml$^{-1}$ respectively (1.5 $\times$ 10$^{-10}$ M and 1.58 $\times$ 10$^{-10}$ M), and that this activity is partially reduced by chemical conjugation to Mint5, being 13.9 and 13.2 ng ml$^{-1}$ respectively (5.6 $\times$ 10$^{-10}$ M and 4.0 $\times$ 10$^{-10}$ M, referred to RIP). Unconjugated Mint5 alone does not affect protein synthesis (data not shown). All functional data taken together indicate that the specific ribosome-inactivating properties of pyramidatine and ocymoidine are substantially preserved in the immunoconjugates.

**Figure 4** Experimental protocols for (**A**) inhibition of proliferation, (**B**) inhibition of protein synthesis. TOX, toxins; IT, immunotoxins; MAb, monoclonal antibody

### Inhibition of cell proliferation and protein synthesis on target cells in vitro

The inhibition of cell proliferation and protein synthesis on A431 target cells was performed in vitro following two different procedures reported as protocols 1 and 2 (Figure 4 A and B). In protocol 1, either toxin, immunotoxin or MAb was added at the time of cell seeding and stayed until the end of the culture, whereas in protocol 2, samples were removed after 1 h of incubation at 4°C followed by 2 h of incubation at 37°C. Figure 5 A shows the effect on A431 cell proliferation of either unconjugated toxin, Mint5 or

**Table 1** In vitro toxicity of Mint–Ocy and Mint–Pyra on different cell lines

| | | Cell proliferation ($IC_{50}$) | | Protein synthesis ($IC_{50}$) | |
|---|---|---|---|---|---|
| **Cell line** | **EGFR no. per cell** | **Mint–Ocy** | **Mint–Pyra** | **Mint–Ocy** | **Mint–Pyra** |
| A431 | $2 \times 10^6$ | 5 PM | 1.5 PM | 10 PM | 10 PM |
| SKBR-3 | $9 \times 10^4$ | 5 PM | 12 PM | 55 PM | 15 PM |
| IGR-OV-1 | $4 \times 10^4$ | 10 PM | 7.5 PM | 15 PM | 5 PM |
| MCF7 | $4 \times 10^4$ | 3 NM | 1 NM | 1 NM | 8 NM |

**Figure 5** Inhibition of cell proliferation and protein synthesis on A431 cell line. The results are expressed as the inhibition percentage of cell proliferation or protein synthesis referred to untreated controls. Each point represents the mean and standard deviation (bars) of quadruplicate determinations of [$^3$H]thymidine (**A** and **B**) or [$^3$H]leucine (**C** and **D**) incorporation immunoconjugate according to protocol 1, indicating that the $IC_{50}$ of Mint–Ocy and Mint–Pyra immunoconjugates are $6.9 \times 10^{-12}$ M and $1.8 \times 10^{-12}$ M respectively. The immunoconjugates show lower activities if removed after initial incubation, as in protocol 2, with $IC_{50}$ of $3 \times 10^{-11}$ M and $2.9 \times 10^{-11}$ M (Figure 5B). The inhibitory activities of unconjugated RIPs are also lower in protocol 2 experiments compared with protocol 1. In fact, $IC_{50}$ for both ocymoidine and pyramidatine are $>10^{-8}$ in protocol 2 and 6–9.6 × $10^{-10}$ in protocol 1. These proliferation experiments indicate that the inhibitory activity of unconjugated Mint5, already reported by Tosi et al (1995) can be strongly potentiated when conjugated to RIP.

Figure 5C shows that Mint–Ocy and Mint–Pyra added to the cell culture according to protocol 1 inhibit A431 protein synthesis with an $IC_{50}$ of $10^{-11}$ M, whereas, when added according to protocol 2, $IC_{50}$ values of $10^{-11}$ M and $1.3 \times 10^{-11}$ M, respectively, were obtained (Figure 5D). Analogously to the inhibition of cell proliferation, protein synthesis inhibition given by unconjugated ocymoidine and pyramidatine was lower in protocol 2 experiments with $IC_{50}$ of $2 \times 10^{-7}$ M compared with protocol 1 values of $IC_{50}$ $2 \times 10^{-8}$ M and $1.5 \times 10^{-8}$ M respectively. The results from the above in vitro experiments suggest a different kinetic of action for immunotoxins on inhibiting proliferation or protein synthesis. In fact, maximal inhibition of proliferation requires the presence of immunoconjugate throughout the time of cell culture, whereas maximal inhibition of protein synthesis is reached after only 3 h of exposure at the beginning of the culture. In order to investigate Mint–Ocy and Mint–Pyra toxic activity as a function of different levels of EGFR expression, both proliferation and protein synthesis experiments were performed on A431, MCF7, SKBR-3 and IGR-OV-1 cell lines according to protocol 1 of Figure 4.

Table 1 shows the concentration of immunoconjugates inducing 50% inhibition of cell proliferation or protein synthesis on the different cell lines. These data indicate the absence of correlation between the toxic action of immunoconjugates and the number of EGFRs on the different cells. The specificity of immunotoxins for EGFR-expressing cells was confirmed on a Jurkat ($EGFR^-$) cell line in which the toxic effect of the immunoconjugates was not greater than that of unconjugated toxins (data not shown).

### In vivo experiments

The anti-tumour efficacy of Mint5 immunotoxins was studied by grafting A431 human tumour cells in athymic mice. The doses of immunoconjugates injected were based on the previously determined doses of ocymoidine and pyramidatine killing 50% of mice ($LD_{50}$) resulting in 13.5 mg $kg^{-1}$ and 2.57 mg $kg^{-1}$ respectively.

© Cancer Research Campaign 1997

**Table 2** Tumour growth inhibition in athymic mice

| Drug | Mean body weight before treatment (g ± s.d.) | Mean body weight at end of treatment (g ± s.d.) | Mean tumour weight at end of treatment (g ± s.d.) | Tumour – body weight ratio | Percentage of inhibition |
|---|---|---|---|---|---|
| PBS | 17.1 (± 2.1) | 17.6 (± 2.8) | 0.7 (± 0.3) | 0.0397 | 0 |
| Mint5 | 16.71 (± 1.8) | 19.5 (± 2.8) | 0.73 (± 0.26) | 0.0374 | 7 |
| Mint–Ocy | 16.61 (± 0.9) | 15.4 (± 1.6) | 0.26 (± 0.13) | 0.0168 | 57 |
| Mint–Pyra | 17.33 (± 1.6) | 16.4 (± 1.5) | 0.34 (± 0.34) | 0.021 | 47 |

Body and tumour weights were determined individually during treatment and the medium was calculated from the 24 animals of each group. Standard deviations (s.d.) are indicated in parentheses. The anti-tumour efficacy was estimated by the tumour–body weight ratio at last day of administration. A significant difference ($P < 0.01$) was found between the control groups (PBS and Mint5-treated) and the test groups (Mint–Ocy and Mint–Pyra). Statistical analysis was performed by ANOVA and Tukey tests.

Taking into account that immunoconjugates are generally at least five times more toxic than unconjugated toxins and that the treatment protocol would include five administrations, the immunotoxin dose of each injection was fixed at 1/25 of the $LD_{50}$ for unconjugated toxins. Moreover, in order to obtain low variability of tumour growth among mice, a rather high number of cells was inoculated ($10^7$ per mouse). The treatment with either immunoconjugate, Mint5 or PBS was started at the same time as tumour grafting and was repeated every other day for a total of five injections. Three days after the last injection, the mice were sacrificed because of evident weight loss, and both body and tumour weights were determined. Table 2 data indicate that Mint–Ocy and Mint–Pyra inhibit 57% and 47% of tumour growth respectively, compared with mice treated with PBS. The inhibitory activity of unconjugated Mint5 was only 7%, although the dose administered was three and seven times higher then Mint5 conjugated to Ocy and Pyra respectively. The necroscopic examination of tissues and organs of animals treated with Mint–Ocy and Mint–Pyra immunoconjugates revealed no significant abnormalities, while microscopic examination revealed a chronic inflammation, pigmentation or margination of the cellular cytoplasm in the liver.

### DISCUSSION

With the aim of obtaining selective toxic agents for cancer treatment, many RIPs have been conjugated to carrier molecules capable of delivering them to specific tumour cell populations. Antibodies, usually monoclonals, are the obvious choice for preparing conjugates, but hormones, growth factors and lectins have also been used as carriers for cancer therapy (Lappi et al, 1991; Wawrzynczak et al, 1991). To date, ricin A has been the most frequently used RIP in preparing immunoconjugates, but more recently, several type 1 RIPs, namely gelonin, PAP saporin, momordin, bryodin and barley RIP, have been used to explore possible medical applications in cancer and autoimmune disease therapy, treatment of graft vs host disease, parasite killing, etc. (Barbieri et al, 1993). For in vivo therapy, the linkage between the antigen-binding molecule and the toxin must be sufficiently stable to remain intact until the immunotoxin reaches its target cells and then the toxin must be released. A saporin-containing immunotoxin, prepared as described here, has been used previously in a clinical trial for the treatment of refractory Hodgkin's disease and proved to be very promising (Falini et al, 1992). Present results indicate that Mint–Ocy and Mint–Pyra immunoconjugates exhibit in vitro tumour cell recognition and inhibition of cell proliferation and protein synthesis. Moreover, the amount of conjugated RIPs required to obtain 50% inhibition ranges from 1:100 to 1:10 000 the amount of unconjugated toxins, indicating that, under different experimental conditions, RIPs' action can be made highly specific and potent. The preliminary in vivo study in tumour-grafted nude mice treated with Mint–Ocy or Mint–Pyra indicates that immunoconjugates are able to control tumour progression efficiently even when a high number of A431 tumour cells is inoculated ($10^7$ per mouse compared with other studies in which A431 cells were inoculated at $10^6$ per mouse) (Baselga et al, 1993). The conditions of treatment for in vivo experiments were fixed in order to achieve the maximum effect of immunoconjugates against a high number of grafted tumour cells. The adopted dose regimen induced weight loss in treated animals, probably owing to liver toxicity; thus, further pharmacokinetic and toxicological studies will be necessary to establish more controlled treatment protocols. Both in vitro and in vivo results indicate that an anti-tumour activity can be obtained by Mint5/RIP conjugate. It is known that toxins and murine antibodies induce an immune response in treated patients (Frankel et al, 1995). Therefore, the identification of new RIPs together with antibody 'humanization' procedures is particularly important in enlarging the list of immunotoxins for clinical use.

Ocymoidine and pyramidatine were previously found to be immunologically cross-reactive only with RIPs from some plants belonging to the same Caryophyllaceae family, but not with RIPs from other plants (Bolognesi et al, 1995). Data from our laboratory (not shown) indicate that, in spite of high N-terminal sequence homology, Ocy and Pyra exhibit only 7.5% of reciprocal cross-reactivity, suggesting their possible use in therapy with sequential immunotoxin treatments. In addition to ocymoidine and pyramidatine, other RIPs are in preparation in our laboratory in order to overcome the immunogenicity problems related to therapeutic regimens requiring multiple administrations. Moreover, a humanized version of Mint5 antibody has been obtained recently in our laboratory showing substantially the same properties of parental MAb (Ferrer et al, 1997).

The EGFR overexpression in some tumour cells compared with normal tissues has been indicated previously as an operational marker for tumour therapy with anti- EGFR monoclonal antibodies (Ennis et al, 1991). Tosi et al (1995) show that Mint5 inhibits the proliferation of tumour cell lines to the same extent despite the difference in EGFR levels. The lack of quantitative discrimination of the levels of EGFR on target cells is also reported in the present paper with Mint–Ocy and Mint–Pyra immunoconjugates. In contrast to our observation, some immunoconjugates with ricin

© Cancer Research Campaign 1997 

toxin have been reported to be able to induce a stronger in vitro inhibitory activity on those cell lines overexpressing EGFR (Masui et al, 1989). Since, from our in vitro inhibitory experiments, the IGR-OV-1 and MCF7 cell lines, which are expressing similar EGFR levels, show different sensitivities to the toxic action of immunoconjugates (Table 1), we believe that in vitro systems are not fully predictive of the biological effects and toxicity of these products in in vivo treatment. In the direction of specific targeting of toxins to EGFR of tumour cells, the generation of immunotoxins directed against an oncogenic mutant of EGFR has been published recently (Lorimer et al, 1995). Mutated EGFR has been identified on gliomas, breast and lung carcinomas, and monoclonal antibodies directed against this mutant do not recognize the wild-type receptor expressed on normal cells.

In general, the toxicity data obtained from clinical trials with immunotoxins induce caution for their systemic use (Frankel et al, 1995).

Possible therapeutic applications of Mint–Ocy and Mint–Pyra immunoconjugates could include the locoregional treatment of brain and bladder cancers. A recent communication reports successful treatment of glioblastoma with immunotoxins directed against the transferrin receptor (Laske et al, 1995). Taking into consideration that glioblastoma cells overexpress EGFR, locoregional treatment with Mint–Ocy and Mint–Pyra immunoconjugates of residual tumour cells after surgery could also be considered. In addition, for this particular application, the induction of immune response would be prevented by the haematoencephalic barrier compartmentalization of immunoconjugates. Since it is generally accepted that recombinant immunotoxins are more stable and more efficient in tumour penetration compared with chemical immunoconjugates (Friedman et al, 1993), the genes coding for both ocymoidine and pyramidatine have been isolated in Menarini Laboratories, and their expression in *E. coli* is in progress either alone or in combination with Mint5 single-chain Fv to obtain recombinant single-chain immunotoxins.

### ACKNOWLEDGEMENTS

Mint5 antibody was obtained under a contract from the National Program of Pharmacological Research (Rif. 078606–1142/188), provided by the Italian Consortium for Anti-tumoral Vectors (CIVA) for the Italian Ministry of University and Scientific and Technological Research. The authors thank Dr Silvana Canevari and Dr Sylvie Ménard of the Istituto Nazionale per lo Studio e la Cura dei Tumori, Milan for helpful discussions and colleagues from SUDBIOTEC S.c.r.l. for the preparation of Mint5.

### REFERENCES

Barbieri L, Battelli MG and Stirpe F (1993) Ribosome-inactivating proteins from plants. *Biochim Biophys Acta*, **1154/3/4**: 237–282

Baselga J, Norton L, Masui H, Pandiella A, Coplan K, Miller WH and Mendelsohn J (1993) Antitumor effects of doxorubicin in combination with anti-epidermal growth factor receptor monoclonal antibodies. *J Natl Cancer Inst*, **85**: 1327–1333

Bolognesi A, Barbieri L, Abbondanza A, Falasca AI, Carnicelli D, Battelli MG and Stirpe F (1990) Purification and properties of new ribosome-inactivating proteins with RNA *N*-glycosidase activity. *Biochim Biophys Acta*, **1087**: 293–302

Bolognesi A, Olivieri F, Battelli MG, Barbieri L, Falasca AI, Parente A, Del Vecchio Blanco F and Stirpe F (1995) Ribosome-inactivating proteins (RNA *N*-glycosidases) from the seeds of *Saponaria ocymoides* and *Vaccaria pyramidata*. *Eur J Biochem*, **228**: 935–940

Endo Y and Tsurugi K (1987) RNA *N*-glycosidase activity of ricin A-chain. Mechanism of action of the toxic lectin ricin on eukaryotic ribosomes. *J Biol Chem*, **262**: 8128–8130

Endo Y and Tsurugi K (1988) The *N*-glycosidase activity of ricin A-chain. The characteristics of the enzymatic activity of ricin A-chain with ribosomes and with rRNA. *J Biol Chem*, **263**: 8735–8739

Endo Y, Huber PW and Wool IG (1983) The ribonuclease activity of the cytotoxin α-sarcin. *J Biol Chem*, **258**: 2662–2667

Ennis BW, Lippman ME and Dickson RB (1991) The EGF receptor system as a target for antitumor therapy. *Cancer Invest*, **9**: 553–562

Falini B, Bolognesi A, Flenghi L, Tazzari PL, Broe MK, Stein H, Durkop H, Aversa F, Corneli P, Pizzolo G, Barbabietola G, Sabattini E, Pileri S, Martelli MF and Stirpe F (1992) Response of refractory Hodgkin's disease to monoclonal anti-CD30 immunotoxin. *Lancet*, **339**: 1195–1196

Ferrer C, Anastasi AM, Di Massimo AM, Bullo A, Di Loreto M, Raucci G, Pacilli A, Rotondaro L, Mauro S, Mele A and De Santis R (1997) Expression and characterization of a mouse/human chimeric antibody specific for EGF receptor. *J Biotechn* (in press)

Fox SB, Smith K, Hollyer J, Greenal M, Hastrich D and Harris AL (1994) The epidermal growth factor receptor as a prognostic marker: results of 370 patients and review of 3009 patients. *Breast Cancer Res Treat*, **29**: 41–49

Frankel AE, Tagge EP and Willingham MC (1995) Clinical trials of targeted toxins. *Cancer Biol*, **6**: 307–317

Friedman PN, Chace DF, Trail PA and Siegall CB (1993) Antitumor activity of the single-chain immunotoxin BR96 sFv-PE40 against established breast and lung tumor xenografts. *J Immunol*, **150**: 3054–3061

Lappi DA and Barid A (1991) Mitotoxins: growth factor-targeted cytotoxic molecules. *Prog Growth Factor Res*, **2**: 223–236

Laske D, Oldfield E and Youle RJ (1995) Immunotoxins for brain therapy. *Fourth Int Symp Immunotoxins*. South Carolina: USA.

Lorimer IAJ, Wikstrand CJ, Batra SK, Bigner DD and Pastan I (1995) Immunotoxins that target an oncogenic mutant epidermal growth factor receptor expressed in human tumors. *Clin Cancer Res*, **1**: 859–864

Masui H, Kamrath H, Apell G, Houston LL and Mendelsohn J (1989) Cytotoxicity against human tumor cells mediated by the conjugate of anti-epidermal growth factor receptor monoclonal antibody to recombinant ricin A chain *Cancer Res*, **49**: 3482–3488

Ozawa S, Ueda M, Ando N, Abe O, Minoshima S and Shimizu N (1989) Selective killing of squamous carcinoma cells by an immunotoxin that recognizes the EGF receptor. *Int J Cancer*, **43**: 152–157

Stirpe F, Bailey S, Miller SP and Bodley JW (1988) Modification of ribosomal RNA by ribosome-inactivating proteins from plants. *Nucleic Acids Res*, **16**: 1349–1357

Strocchi P, Barbieri L and Stirpe F (1992) Immunological properties of ribosome-inactivating proteins and of a saporin–IgG conjugate. *J Immunol Methods*, **155**: 57–63

Thorpe PE, Wallace PM, Knowles PP, Relf MG, Brown ANF, Watson GJ, Blakey DC and Newell DR (1988) Improved antitumor effects of immunotoxins prepared with deglycosylated ricin-A-chain and hindered disulfide linkages. *Cancer Res*, **48**: 6396–6401

Thrush GR, Lark LR, Clinchy BC and Vitetta ES (1996). Immunotoxins: An update. *Annu Rev Immunol*, **14**: 49

Tosi E, Valota O, Negri D, Adobati E, Mazzoni A Meazza R, Ferrini S, Colnaghi MI and Canevari S (1995) Antitumor efficacy of an anti-epidermal growth factor receptor monoclonal antibody and its $F(ab')_2$ fragment against high and low EGFR-expressing carcinomas in nude mice. *Int J Cancer*, **62**: 643–650

Wawrzynczak EJ (1991) Systemic immunotoxin therapy of cancer: advances and prospects. *Br J Cancer*, **64**: 624–630

Workman P, Balmain A, Hickman JA, McNally NJ, Mitchinson NA, Pierrepoint CG, Raymond R, Rowlatt C, Stephens TC and Wallace J (1988) UKCCCR guidelines for the welfare of animals in experimental neoplasia. *Br J Cancer*, **58**: 109–113

 © Cancer Research Campaign 1997